# The feasibility of mixed reality-based upper extremity self-training for patients with stroke—A pilot study

**DOI:** 10.3389/fneur.2022.994586

**Published:** 2022-09-28

**Authors:** Yeajin Ham, Dong-Seok Yang, Younggeun Choi, Joon-Ho Shin

**Affiliations:** ^1^Department of Rehabilitation Medicine, National Rehabilitation Center, Ministry of Health and Welfare, Seoul, South Korea; ^2^Business Growth Support Center, Neofect, Seongnam, South Korea; ^3^Department of Computer Engineering, Dankook University, Yongin-si, South Korea

**Keywords:** augmented reality, equipment, gamification, mixed reality, telerehabilitation, supplies

## Abstract

Mixed reality (MR), which combines virtual reality and tangible objects, can be used for repetitive training by patients with stroke, allowing them to be immersed in a virtual environment while maintaining their perception of the real world. We developed an MR-based rehabilitation board (MR-board) for the upper limb, particularly for hand rehabilitation, and aimed to demonstrate the feasibility of the MR-board as a self-training rehabilitation tool for the upper extremity in stroke patients. The MR-board contains five gamified programs that train upper-extremity movements by using the affected hand and six differently shaped objects. We conducted five 30-min training sessions in stroke patients using the MR-board. The sensor measured hand movement and reflected the objects to the monitor so that the patients could check the process and results during the intervention. The primary outcomes were changes in the Box and Block Test (BBT) score, and the secondary outcomes were changes in the Fugl–Meyer assessment and Wolf Motor Function Test (WMFT) scores. Evaluations were conducted before and after the intervention. In addition, a usability test was performed to assess the patient satisfaction with the device. Ten patients with hemiplegic stroke were included in the analysis. The BBT scores and shoulder strength in the WMFT were significantly improved (*p* < 0.05), and other outcomes were also improved after the intervention. In addition, the usability test showed high satisfaction (4.58 out of 5 points), and patients were willing to undergo further treatment sessions. No safety issues were observed. The MR-board is a feasible intervention device for improving upper limb function. Moreover, this instrument could be an effective self-training tool that provides training routines for stroke patients without the assistance of a healthcare practitioner.

**Trial registration:**

This study was registered with the Clinical Research Information Service (CRIS: KCT0004167).

## Introduction

Patients with stroke often suffer from upper limb dysfunction, which impedes their activities of daily living and quality of life (1). Much rehabilitation is necessary to obtain meaningful recovery of the upper extremities, but access to such intervention is limited. Typically, only a short duration of inpatient rehabilitation is permitted, and time for rehabilitation in an outpatient rehabilitation setting is limited. Consequently, from the perspective of a continuum of rehabilitation, home-based rehabilitation is essential, as it allows continuous and sufficient rehabilitation over a longer period of time, eliciting functional improvement (2). The patients' difficulties in accessing rehabilitation facilities in terms of mobility, transport, or caregiver issues also highlight the importance of home-based rehabilitation or self-training (3).

However, there are currently barriers to the widespread use of home-based rehabilitation, as limited resources are available: human resources, such as physical or occupational therapists, are insufficient to provide in-person interventions. Therefore, an alternative method has been investigated, and the use of newly developed instruments, such as games, telerehabilitation, robotic devices, virtual reality (VR) devices, sensors, and tablets have been tested (2). Additionally, a recent study demonstrated the non-inferiority of dose-matched home-based rehabilitation using technology compared with in-clinic therapy (4). However, most of these technologies are not available in the real world because of safety issues, the need for the assistance of healthcare practitioners, or cost. Considering these barriers, VR-based rehabilitation might play a promising role in telerehabilitation (5).

VR technologies limit the input interface for hand rehabilitation because the hand plays a substantial role as an end-effector of the upper extremity, and as such is crucial to activities of daily living and quality of life (6, 7). The hand is a complex and versatile structure with a high degree of freedom as compared to the proximal upper limb. Thus, adopting VR technology is challenging for applying meticulous movement of the affected hand. Commonly used types of hand rehabilitation involve wearing of devices or sensors. A robotic device with a rigid body can be used for rehabilitation in medical facilities; however, it is difficult to use it at home without healthcare professionals because of wearing or safety issues. Therefore, commonly used input interfaces are flexed wearable devices, such as the RAPAEL Smart Glove^™^ (Neofect, Yong-in, Korea) and HandTutor (MediTouch, Netanya, Israel) (8, 9). However, these wearable glove-type devices have some drawbacks: they do not fit all hand sizes, the weight and size of the device affect the training, the problem with hygiene, and limited range of motion due to the device (10, 11). On the other hand, a depth camera-based system [e.g., Xbox^™^ Kinect (Microsoft, Redmond, WA, USA)], which is commonly used in rehabilitation, is also not ideal for hand rehabilitation because of the difficulty in reflecting minute hand movements (12). Additionally, these input devices lack sensory feedback from the hands because they typically involve manipulating virtual objects, rather than tangible objects. A recent study suggested that training with actual objects might augment interactivity with tactile sense, as compared to training with virtual objects, which utilize only the visual sense (13).

To address these issues, we developed a mixed reality (MR)-based rehabilitation board (MR-board) that adopts a depth sensor-based VR as well as tangible objects for rehabilitation of the upper limb, and particularly for hand rehabilitation. MR refers to a user space stemming from the merging of virtual and real worlds, which enables real-time interactions between physical and virtual objects (14). Considering the introduction of this novel technology, we here conducted a feasibility study and the usability of the MR-board for upper extremity rehabilitation, including that of the hand. Furthermore, we assessed the potential to train functional activities for patients with upper limb impairment after a stroke as self-training tool.

## Materials and methods

The institutional review board of our rehabilitation hospital approved this study (NRC-2018-01-006), and all patients provided written informed consent before enrollment. All subjects gave written informed consent in accordance with the Declaration of Helsinki.

This study was performed with a single-group pre–post design, in a research room in a single rehabilitation hospital. In preparation for home-based self-training, the research environment was set like a home. The subjects were encouraged to exercise their upper limb in an individual room separated from the therapist with minimal regulation, and the therapist did not intervene during the training in preparation for self-training. Also, the room was furnished with furniture such as a refrigerator and a sofa to resemble a house.

### Patients

The inclusion criteria were as follows: (i) age >19 years; (ii) diagnosis of first-ever hemispheric stroke that resulted in unilateral upper limb functional deficits, as identified from the medical records; (iii) cognitively capable of understanding and following instructions (Mini-Mental State Examination score ≥ 24) (15); and (iv) Brunnstrom motor recovery level of 4 of semi-voluntary finger extension in a small range of motion, and lateral prehension with release by the thumb in the affected arm and hand (16). The exclusion criteria were as follows: (i) bilateral brain lesions, (ii) any neurological disorder other than stroke, (iii) any other severe medical condition, and (iv) predisposing severe pain in the upper limb that could impede training.

### Apparatus

#### Instrument description

**Figure 1:**
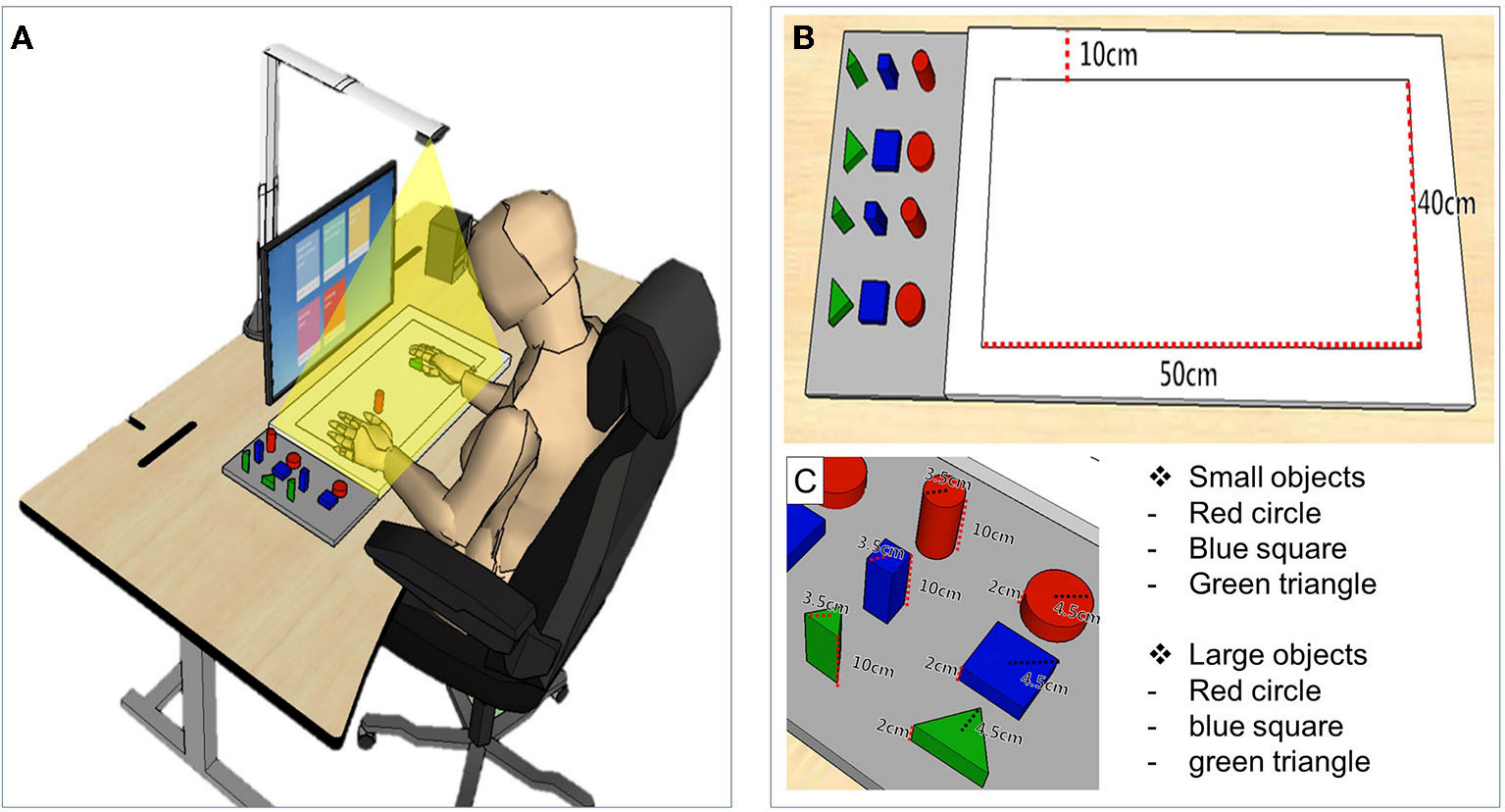
The simulation of training and description of detailed devices in mixed reality (MR)-board. **(A)** Schematic illustration of training using the MR-board. The camera on the MR-board displayed all movements of the hands and objects on the board on the monitor. **(B)** Specifications of the MR-board. The thickness of the board was 10 cm and the actual training space was 500 mm (length) × 400 mm (width). **(C)** The objects consisted of two sets of six shapes and sizes.

The MR-board was developed to improve upper limb function, focusing on the hand. The MR-board consists of a board plate, depth camera, shaped objects made of plastic, and a monitor (Figure 1). The board plate was 700 mm (length) × 600 mm (width), with a thickness of 100 mm, and weight of 22 lb. The depth camera (Intel^®^ Realestate^™^ SS300 Camera, Intel Realestate, Santa Clara, CA, USA) that illuminated the board plate was suspended above the board plate to detect the patient's hand movement. The specifications of the camera were 12.5 mm (length) × 110 mm (width) × 3.75 mm (height). The tangible objects consisted of three different shapes (triangle, square, and circle) and colors (red, blue, and green) with two different sizes: large [35 mm (diameter) × 100 mm (height)] and small [45 mm (diameter) × 30 mm (height)]. A 24-inch monitor, which was placed 700 mm in front of the patients, reflected the real-time motion of the virtual object.

#### Technology used in system interface

The detection of the ground and boundary of the board plate and of objects was performed based on the analysis of color and depth images. The boundary was set using the Harris corner detector, and affine transformation was used to correct distortion (17). For equalization, we applied the median value to all depth values (we set the depth value of the performance area at zero). The function of the algorithm is given by:


(1) $$\begin{array}{l} d_{normal}\;=\;d_{input}\\ \;-\;d_{median\;of\;work\;space}\;(R>\;\omega idth,\;\;height)\; \end{array}$$

In addition, the HSV color model was applied to distinguish between objects and hands (18), and Gaussian blur and morphology methods were applied to remove any noise (19). The objects were distinguished in detail by using color and depth information. The object size (large or small) was determined using the average depth value of the object. Setting the depth value of the board plate to 0, the depth values of the large and small sizes were 100 and 20, respectively. We used the RGB color model to recognize colors and the convex hull algorithm to recognize shapes (20). Additionally, we adopted a lightweight deep-learning-based hand geometric feature extraction and hand position estimation method to reflect delicate hand movements.

#### Contents of training programs

**Figure 2:**
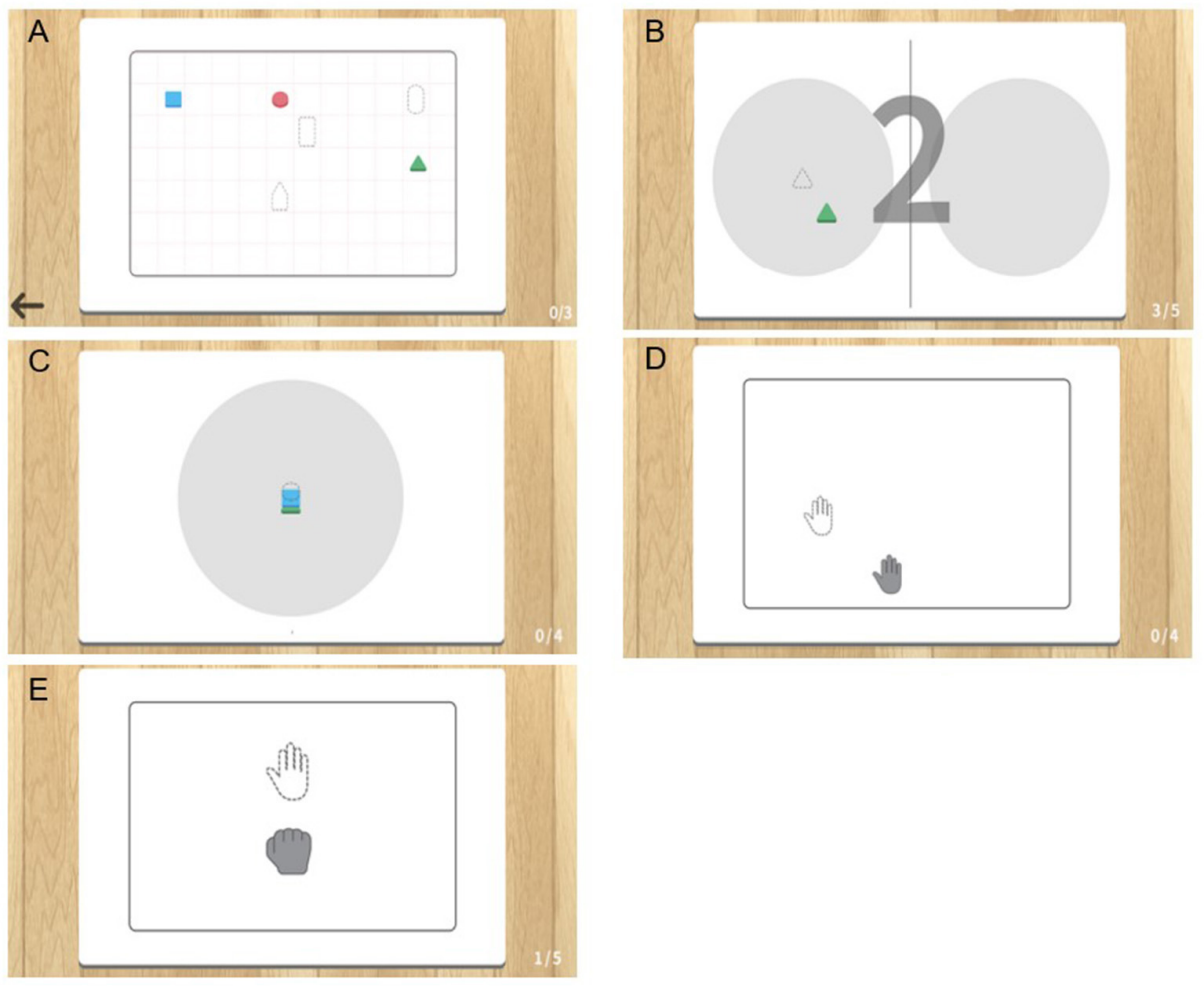
Illustration of five gamified programs included in the mixed reality (MR)-board. **(A)** Matching the same shape. **(B)** Moving the object. **(C)** Stacking the objects. **(D)** Placing the arm. **(E)** Grasp and release.

**Figure 3:**
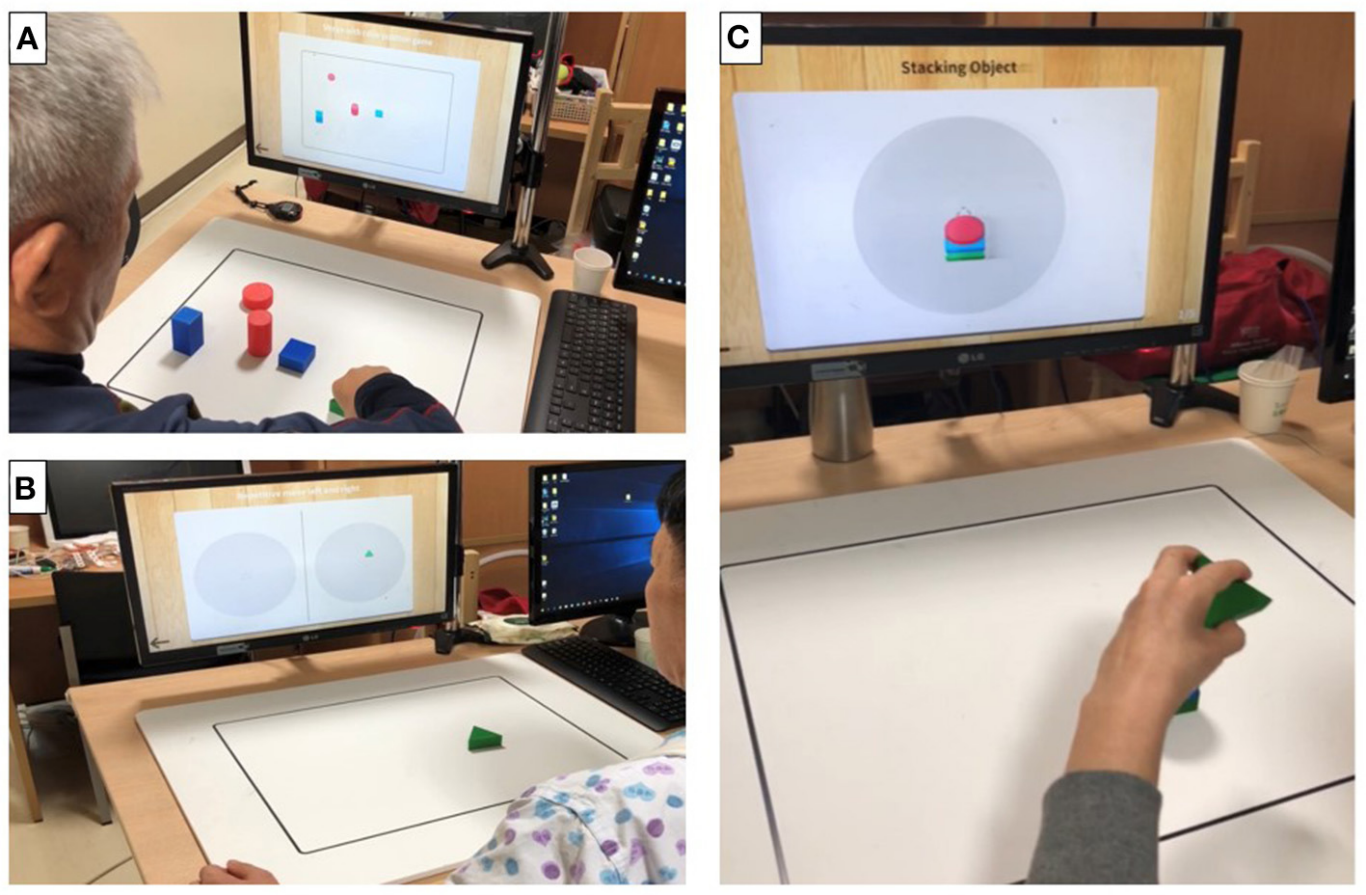
Photographs of actual training using the objects in mixed reality (MR)-board training. The photographs show actual training using objects. Visual feedback was provided to the patients through a monitor that reflected the position of the object on the board. **(A)** “Matching the same shape” involved positioning of six objects correctly while comparing those displayed on the monitor. **(B)** “Moving the object” involved moving specific objects to the right and left repetitively. **(C)** “Stacking the objects” involved stacking objects on top of one another, without them collapsing, according to the shape shown on the monitor.

The MR-board included five games that originated from commonly performed self-training tasks in the real world. The tasks were mainly based on reaching and grasping, which are fundamental skills for many daily activities and are prerequisites for complex fine movements, and have therefore been a significant target in upper limb rehabilitation (21). In three games, patients were asked to use the shaped objects, and two games were performed using the hands without the objects. Object-detection techniques were used for the tasks in which the shaped objects were used. Object detection techniques provided information on object location and category by detecting objects in particular classes (hand and background) using digital images including photos and videos (22). Each game program is described below and is illustrated in Figure 2. Photographs of the actual training are shown in Figure 3.

- “Matching the same shape” was inspired by pegboard training. Patients placed shaped objects on the board plate, matching the location and shape reflected on the monitor. The goal was to place the appropriate object in the correct place, for all six objects. Patients could finely modify the position of the objects based on the feedback of the results reflected on the monitor. The outcome was the total performance time required to complete the task.
- “Moving the object” came from the box and block test (BBT). Patients grasped and moved objects from right to left or vice versa, repeatedly following the written instructions shown on the monitor. The outcomes were the number of objects moved and the time required to complete each object.
- “Stacking the objects” stemmed from stacking cones. Patients were asked to stack objects in the same order as they appeared on the monitor. Patients could change the difficulty level by setting the number of objects stacked from a minimum of three to a maximum of six. The outcomes were the number of objects used and the time required to complete the task.
- The “Placing the arm” task required patients to place the affected arm in targeted points suggested on the monitor and to maintain the position for 5 s. Patients could program the system with the number of times they would like to repeat the practice before starting the task. The outcomes were shown as the number of a hand moved and the total performance time to complete the tasks.
- The “Grasp and release” task involved repeated grasp and release actions. The patients were asked to mimic the movement reflected on the monitor, and holding the required position for 3 s. The patients could program the system with the number of times they would like to repeat the practice before starting the task. The outcome was the performance time, which was defined as the number of times the set was completed.

### Procedures

All patients received five 30-min training sessions (5 days per week) using the MR-board in the research intervention room. Before starting the intervention, an experienced occupational therapist provided brief instructions, including an explanation of the purpose of the study and descriptions of the five training programs. The patients trained their upper extremities by themselves, following instructions provided by the system. During the training, the therapist did not intervene; instead, she supervised only for potential safety issues and aided patients when they needed assistance in controlling the equipment. Thus, the therapist was seated in a space separated by a wall from the area in which the patients trained and only appeared when called by the patient. The training process was reflected automatically on the monitor, giving the patient visual feedback.

Ten patients completed five programs every day, with each program lasting ~5 min. The patients adjusted the difficulty level of the program by setting the target number for each task (e.g., number of shaped objects moved, or number of grasps and releases performed) or the total performance time. The difficulty level was changed by clicks of the mouse before starting the program.

### Outcome measures

An experienced occupational therapist assessed the outcome measures. Evaluations were conducted twice: pre- and post-intervention. The pre-test was performed the day before the first day of the intervention, while the post-test was performed the day after the final day of the intervention. Demographic characteristics were recorded and included sex, age, affected side of paresis, and time from stroke.

#### Primary outcome

The primary outcome was the change in BBT scores. The BBT measures unilateral gross manual dexterity by counting the number of blocks that can be moved from one compartment to another within 1 min (23).

#### Secondary outcomes

The secondary outcomes were the change in the Fugl–Meyer Assessment Upper Extremity (FMA-UE) score and the Wolf Motor Function Test (WMFT). The FMA-UE is a stroke-specific, performance-based quantitative measure of upper limb motor impairment, with a higher score indicating lesser motor impairment (24). The FMA-UE consists of 33 items, ranging from 0 to 66. The WMFT is an upper extremity assessment tool that uses timed and functional tasks (25). The WMFT consists of 17 tasks, 15 of which are functional and two of which are strength-related, measuring shoulder and grip strength (26). The total score on the functional ability scale for items was assessed on a six-point scale (WMFT-score; higher scores indicate better motor function), and the total amount of time for each item (WMFT-time; shorter time indicates better performance) (27).

After completing five sessions of MR-board training, we asked patients to complete a usability test for the device, using both 5-point Likert scale–based questionnaires and interviews. Questionnaires assessed usability regarding MR-board training in eight domains, with a higher score indicating higher satisfaction: specification, weight, manipulation, safety, durability, friendliness, comfort, and effectiveness. Additionally, we asked each patient the following question: “Are there any other comments that you want to give us, such as which aspects were good or bad, or any recommendations for future development of the device?” We aggregated personal opinions about using the MR-board as a training tool.

#### Statistical analysis

Patients who completed all the sessions of the designated intervention were included. Wilcoxon signed-rank tests were used to compare the repeatedly measured clinical scales and tests, including the BBT, FMA-UE, and WMFT. Furthermore, we indicated the effect sizes to interpret the results (Cohen's *d*) and calculated the standardized mean difference of performance changes. Cohen's *d* was interpreted as follows: 0.2–03 represented a small effect size, 0.5 represented a medium effect size, and ≥ 0.8 represented a large effect size (28). Statistical analysis was performed using R 4.1.3 (http://www.r-project.org; R Foundation for Statistical Computing, Vienna, Austria). Statistical significance was set at *p* < 0.05.

## Results

### Participant characteristics

Ten patients with hemiplegic stroke (2 women; mean ± standard deviation age: 55 ± 7.4 years) were included in this study (Table 1). The average time from onset was 30.5 ± 26.9 months (minimum–maximum: 1–79 months). Three patients were in the subacute stage (<6 months post-onset) (29).

**Table 1:** Demographic and clinical characteristics of the patients.

**ID**	**Sex**	**Age**	**Duration** **since the** **stroke** **(months)**	**Affected** **side** **(hand)**	**Type of stroke**	**MMSE**	**Admission** **status**	**BS** **(distal)**	**MRC-** **elbow** **(Fl/Ex)**	**MRC-** **wrist** **(Fl/Ex)**	**MRC-** **finger** **(Fl/Ex)**
1	M	57	11	Left	Right thalamus ICH	28	Inpatient	5	4/3	4/4	4/4
2	M	55	63	Right	midbrain, pons ICH	27	Outpatient	5	4/3	4/4	4/3
3	M	53	41	Left	Right thalamus ICH	29	Outpatient	4	3/3	4/3	3/3
4	M	60	79	Right	Cerebral infarction	30	Outpatient	5	4/3	4/4	4/3
5	M	65	3	Right	Basal ganglia ICH	28	Inpatient	5	4/4	4/4	4/4
6	M	38	4	Left	arteriovenous malformation	29	Inpatient	5	4/4	4/4	3/3
7	M	63	1	Right	pontine hemorrhage	26	Inpatient	6	4/4	4/4	4/4
8	F	54	24	Left	Right brain hemorrhage	30	Outpatient	5	3/3	3/3	3/3
9	F	52	34	Right	Cerebral infarction	29	Outpatient	6	4/4	4/4	4/4
10	M	53	45	Left	Rt. thalamus ICH	29	Outpatient	5	4/4	4/4	4/4

BS, Brunnstrom stage; Ex, extensors; F, female; Fl, flexors; ICH, Intracerebral hemorrhage; M, male; MMSE, Mini-Mental State Examination; MRC, Medical Research Council Scale for Muscle Strength.

### Outcome measurements

**Figure 4:**
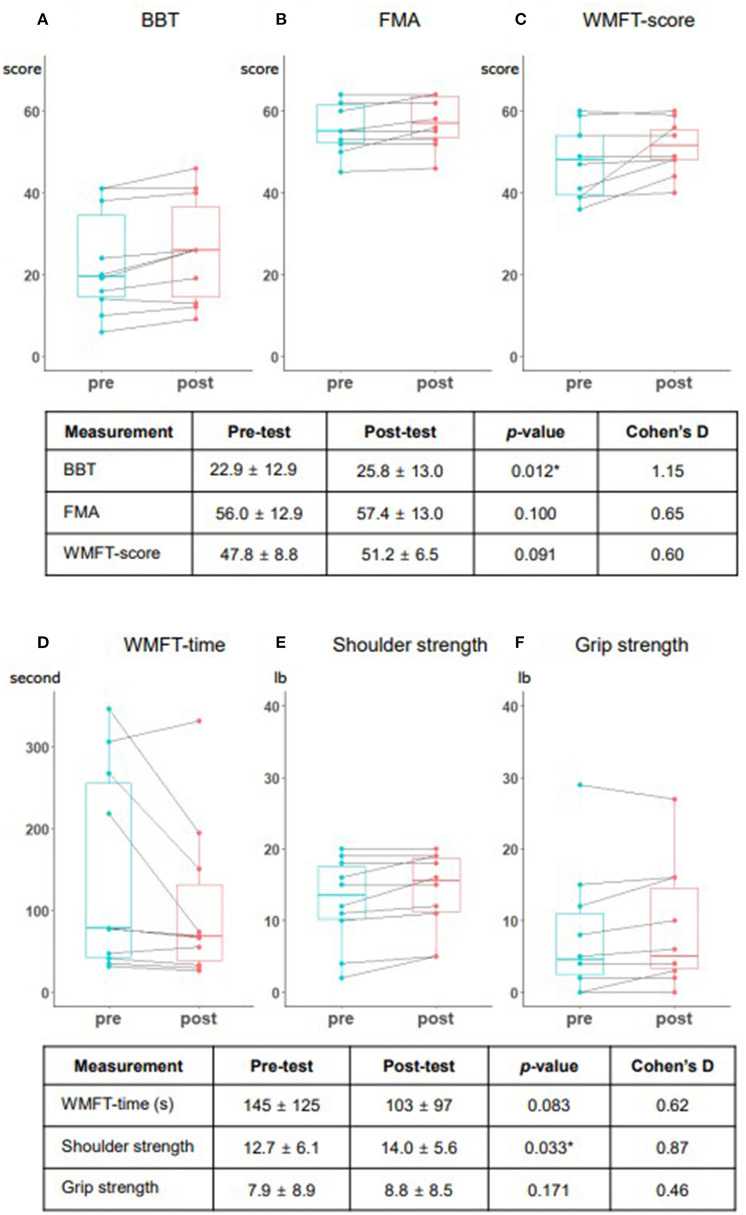
Performance changes among the study patients presented in box plots. The box plots present the results of each outcome measurement and individual change. **(A)** Box and block test (BBT). **(B)** Fugl-Meyer assessment (FMA). **(C)** Wolf Motor Function Test (WMFT) score. **(D)** WMFT time. **(E)** Shoulder strength. **(F)** Grip strength. Each measurement includes the mean and standard deviation of the pre- and post-test, the *p*-value based on the Wilcoxon signed-rank test, and Cohen's D score. BBT and shoulder strength were statistically significant (*p* < 0.05).

The results of outcome measurements and each patients' improvement are shown in Figure 4 as box plots. After five sessions of interventions, the primary outcome (BBT) showed a large effect size and statistically significant average, from 22.9 to 25.8 scores (Cohen's *d* = 1.15, *p* = 0.012); while secondary (FMA-UE, WMFT) outcomes showed improving trends, with medium effect sizes, although the changes were not statistically significant. In the WMFT subtest, shoulder strength also showed significant improvement, with a large effect size (Cohen's *d* = 0.87, *p* = 0.033). There were no occurrences of falls or shoulder pain observed or reported during all training sessions, and there were no adverse events such as skin problems, thereby confirming safety. Moreover, the usability test showed a high satisfaction level, with a mean of 4.58 ± 0.54 out of five points. Safety and comfort achieved the highest scores, while effectiveness achieved the lowest score (Table 2). We conducted additional interviews to gather personal opinions, as summarized in Supplementary Table 2. We collapsed the responses into four components: hardware system in the MR-board, gamification, training methods, and self-training tool aspects. In addition, we classified responses as positive feedback, negative feedback, or suggestions.

**Table 2:** Usability test of the mixed reality-based rehabilitation board (MR-board) in eight domains.

**Component**	**Question**	**Score**
Specification	Was the size, height, depth, and width of the MR-board appropriate?	4.5 ± 0.71
Weight	Was the weight of the device and objects appropriate?	4.2 ± 0.79
Manipulation	Was it convenient to adjust the components of the device?	4.8 ± 0.63
Safety	Is the MR-board safe and robust to use?	5.0 ± 0.00
Durability	Do you think the MR-board will be available for a long time?	4.5 ± 0.71
User friendliness	Was the MR-board easy to use?	4.6 ± 0.84
Comfort	Was it comfortable to use the MR-board?	5.0 ± 0.00
Effectiveness	Was the MR-board helpful for upper limb rehabilitation?	4.0 ± 0.67
Total average		4.58 ± 0.54

The test was rated on a 5-point scale ranging from 1 (very dissatisfied) to 5 (very satisfied).

Data are presented as mean ± standard deviation.

## Discussion

In this pilot study, we newly developed an MR-based device targeted for self-training of upper extremities in stroke patients. Within the five training sessions, the patients showed increasing trends in outcome measurements, including statistical improvements in the BBT score and shoulder muscle strength of the WMFT. Training that is similar to the movements of BBT might affect the outcome measurements. Still, the statistical difference and trend of improvement in all outcome measurements in such a short period were still meaningful since the BBT and WMFT assessed the functional activities of the patients. Additionally, its patient-rated usability scores were high. In particular, the increased adherence and safety, which was evidenced by the lack of drop-outs and of adverse events during the study, emphasize the possibility of using the MR-board as a self-training rehabilitation tool. Upper extremity training using the MR-board resulted from the useful features of each component of our system. First, we used the input interface as a single-depth camera, not requiring wearing of or contact with the device, which might impede tactile input and range of motion, and which reflected the movement of the training hand (10, 11). Previous studies using camera sensors had some limitations. One study used RGB-D to apply convolutional neural networks to localize hand movement, but had difficulty determining occlusion when using a single camera (30). Other studies used multiple cameras to sense the motion and effectively collect data without a blind spot; however, they required more space and time to install the cameras (31, 32). In addition, these studies focused on collecting hand motion data, but did not develop programs for hand training as part of rehabilitation. Our MR-board used a single camera that could be installed simply and rapidly, without requiring much space. In addition, our system classified the hand and object and reflected hand movement by applying lightweight deep-learning-based hand-geometric feature extraction and hand-position estimation methods, causing less occlusion.

Second, our system employed various tangible shaped objects to manipulate a virtual object, which acted as a “tangible user interface” (33). These tangible interfaces enable a “MR” experience, leading to active participation and effective and natural learning (34). The haptic sense obtained by grasping and manipulating real objects allowed patients to obtain sensory information that incorporated tactile sensory, depth, and spatial data and facilitated realistic and explicit experiences (13, 35). Considering that the visual and auditory senses are virtual, the real haptic sense may be integral to the MR system (36). Thus, our MR-board, which incorporated VR and sensory input, could elicit plasticity more than other interfaces. In addition, reaching and grasping patterns are known to change depending on the properties of the target objects; thus, the diverse shapes and sizes of the objects used might enhance coordination ability across various situations (37).

Third, the MR-board contained five gamified programs to encourage the repetitive movement of the affected hand. Our program originated from conventional rehabilitation tools, such as stacking cones, the box and block test, and pegboards, which are widely used in traditional rehabilitation settings. Conventional rehabilitation lacks a fun element, which makes patients bored and lowers their adherence. Thus, gamified rehabilitation instruments, which incorporate computerized technologies and traditional rehabilitation tools, have been investigated (13, 38). In line with previous studies, we developed MR-board programs, which adopted traditional upper limb training tools, including hand movement training, and added gamified systems involving scoring, sensory stimuli, challenges, and therapy levels. These gamified programs facilitate repetitive activity by reflecting the hand movements, allowing the patient to concentrate more on the training.

Taken together, the virtual hand and objects were reflected in the display, synchronized with the real hands and objects, without the need to wear any device. Thus, the patients reported an understanding of their upper-limb movement more than under conventional training conditions. In addition to the realistic feeling, the gamified content allowed greater immersion of patients and enhanced their adherence to the training. Therefore, these technical components of our MR-board enabled repetitive training by allowing patients to be immersed in a virtual environment, while maintaining the perception of the real world, leading to clinical effectiveness. These reports reflected the high adherence rate, shown by the lack of drop-out during the intervention, and by the positive usability test results. High adherence is affected by increased independence, experience of health benefits, and motivation maintenance, which are essential for long-term home-based rehabilitation (39). Therefore, maintaining a high adherence rate using the MR-board would allow patients to adhere to their upper extremity training independently.

In addition, the MR-board has many strengths as a home-based self-training tool in the future. The usability test showed that the safety and comfort of the MR-board were sufficient for a home-based rehabilitation instrument for stroke patients. In this study, all patients were trained independently, with no therapist involvement during MR-board training. The research therapist only set programs at the start of the training, while the patients were programmed the system during the intervention. In addition, the easy and user-friendly aspects of the MR-board were great merits for the patients in terms of simple installation, concise instruction, and ease of changing the difficulty level. Finally, not much space was needed to install the device. These properties of the MR-board would allow stroke patients to preserve function without needing to visit hospitals for rehabilitation training, by facilitating at-home self-training.

This study has several limitations. First, it was a single-arm study with no control group, rather than a randomized controlled trial. A single-arm study has reduced internal validity and reduced intervention effectiveness, and do not account for confounding factors, such as spontaneous recovery (40). Second, five training sessions were insufficient to improve patients' upper extremity function. We found an increasing trend in all outcome measurements after training; however, more intervention periods should be used in future studies. Third, we originally planned to collect the data on daily performance of the MR-board system; however, we could not retrieve the data on the results due to technical errors in the MR system. An upgraded version of the MR-board is needed to store the daily results safely and visually. Forth, bias may have been caused by patients who remained functional with mild deficits, i.e., the ceiling effect (41). The patients in our study were limited to those with mild upper extremity deficits; all but one patient had a Brunnstrom stage of 5 or 6 in the distal part. Therefore, the program might have been too easy for them, and they needed more complex training programs for their hands and fingers. Patients also asked for more versatile objects for training, rather than limiting them to specific materials, sizes, and weights. In a future study, we plan to add more programs for training individual fingers and a mini camera to prevent occlusion. Fifth, the results could have been influenced by the usability of the data. Patients may not have felt comfortable in directly criticizing or expressing dissatisfaction with the device in the verbal interviews. However, even after accounting for these factors, the satisfaction levels remained high and positive.

## Conclusion

We developed the MR-board as an upper extremity training tool for patients with stroke and applied deep learning algorithms to sense hand movements using a camera and tangible objects for upper extremity rehabilitation using an MR environment. We conducted small sessions of the intervention to test the feasibility of the MR-board. We also conducted a usability test of the device that can be used as a rehabilitation instrument for patients with stroke and showed its potential as an effective self-training rehabilitation tool.

## Data availability statement

The raw data supporting the conclusions of this article will be made available by the authors, without undue reservation.

## Ethics statement

The studies involving human participants were reviewed and approved by Institutional Review Board in National Rehabilitation Center. The patients/participants provided their written informed consent to participate in this study. Written informed consent was obtained from the individual(s) for the publication of any potentially identifiable images or data included in this article.

## Author contributions

D-SY and YC conceived of the present idea and developed the MR-board. YH implemented the training program and wrote the manuscript in consultation with J-HS. Finally, J-HS designed and verified the analytical methods and supervised the findings of the work. All authors contributed to the article and approved the submitted version.

## Funding

This work was supported by the Technology Innovation Program (or Industrial Strategic Technology Development Program-Technology Innovation Program) (20014480, A Light-Weight Wearable Upper Limb Rehabilitation Robot System and Untact Self-Training and Assessment Platform Customizable for Individual Patient) funded by the Ministry of Trade, Industry and Energy (MOTIE, Korea) and the National Research Foundation of Korea (NRF) grant funded by the Korea Government (MSIT) (NRF-2021R1A2C2009725, Development of Wearable Camera Based VR Interface Using Lightweight Hand Pose Estimation Deep Learning Model).

## Conflict of interest

Author D-SY was employed by Neofect. The remaining authors declare that the research was conducted in the absence of any commercial or financial relationships that could be construed as a potential conflict of interest.

## Publisher's note

All claims expressed in this article are solely those of the authors and do not necessarily represent those of their affiliated organizations, or those of the publisher, the editors and the reviewers. Any product that may be evaluated in this article, or claim that may be made by its manufacturer, is not guaranteed or endorsed by the publisher.
