# Supplementary material for: The feasibility of mixed reality-based upper extremity self-training for patients with stroke—A pilot study
Source: Front Neurol. 2022 Sep 28;13:994586. doi: 10.3389/fneur.2022.994586 (PMC9555565; doi:10.3389/fneur.2022.994586)
Supplement: Supplementary file 1 [file Table_1.DOCX]

**Supplementary Table 1. Performance changes among the study patients**

| Variable | Patients (n = 10) | | | |
| --- | --- | --- | --- | --- |
|  | Pre-test | Post-test | P-value | Cohen’s d |
| BBT | 22.9 ± 12.9 | 25.8 ± 13.0 | 0.012* | 1.15 |
| FMA-proximal | 32.0 ± 3.4 | 32.8 ± 3.4 | 0.094 | 0.51 |
| FMA-distal | 22.0 ± 2.2 | 22.5 ± 2.1 | 0.371 | 0.46 |
| FMA-coordination | 2.0 ± 1.6 | 2.1 ± 1.7 | 0.343 | 0.32 |
| FMA-total | 56.0 ± 12.9 | 57.4 ± 13.0 | 0.100 | 0.65 |
| WMFT-score | 47.8 ± 8.8 | 51.2 ± 6.5 | 0.091 | 0.60 |
| WMFT-time (s) | 145 ± 125 | 103 ± 97 | 0.083 | 0.62 |
| Shoulder strength | 12.7 ± 6.1 | 14.0 ± 5.6 | 0.033* | 0.87 |
| Grip strength | 7.9 ± 8.9 | 8.8 ± 8.5 | 0.171 | 0.46 |

Abbreviations: FMA, Fugl–Meyer assessment; BBT, Box and Block Test; WMFT, Wolf Motor Function Test

Data are presented as mean ± standard deviation.

Wilcoxon signed-rank test was performed

* Statistically significant
