# Supplementary material for: The feasibility of mixed reality-based upper extremity self-training for patients with stroke—A pilot study
Source: Front Neurol. 2022 Sep 28;13:994586. doi: 10.3389/fneur.2022.994586 (PMC9555565; doi:10.3389/fneur.2022.994586)
Supplement: Supplementary file 2 [file Table_2.DOCX]

**Supplementary table 2. Summarized interviews for usability aspects with the mixed-reality based rehabilitation board (MR-board).**

| Component | Key elements |
| --- | --- |
| Hardware system in the MR-board | - Tangible objects of different shapes incorporated into virtual reality allowed obtaining sensory input and depth and spatial information (Positive feedback). - More varied game programs with upgraded graphical aspects will enhance immersion. Additionally, a variety of levels are required particularly for training of individual finger movements (Suggestion) - More varied objects with different sizes, weights, and tactile input are needed. For example, objects made of wood would give more weight and tactile information than objects made of plastic. Additionally, varied sizes could allow for training more delicate hand and finger movements. (Suggestion) |
| Gamification | - Gamified programs reflecting hand movement in the monitor allowed patients to concentrate more on the training (Positive feedback) - More “fun” components for engagement might be preferred, such as a video game, rather than simple gamified contents, such as scoring or time on the screen. (Suggestion) |
| Training method | - Understanding the movement of the affected hand due to instant visual feedback on the screen (Positive feedback) - In comparison to virtual reality training, training with real objects allows for more realistic and explicit experiences, and it does not produce cybersickness. (Positive feedback) - Since other devices were not worn, natural hand movements can be checked and are free from being affected by the joint range of motion or sensory input (Positive feedback) - More training time might be useful due to the unfamiliar MR-system, thus lengthening the time of a session would be required for the affected upper limb and hand (Suggestion) |
| Home-based self-training | - Easy to install the device, does not require much space for the device (Positive feedback) - The instruction offered by the system was concise and straightforward (Positive feedback) - There were no adverse events observed during the training (Positive feedback) - It is easy to change the difficulty level; a simple click changes the number of objects and training time (Positive feedback) - Periodical tele-supervision from physiatrists or therapists are needed for the supervision or setting the optimal goal for the patients (Suggestion) |
